# Occupational stress and the risk of turnover: a large prospective cohort study of employees in Japan

**DOI:** 10.1186/s12889-020-8289-5

**Published:** 2020-02-04

**Authors:** Yuko Kachi, Akiomi Inoue, Hisashi Eguchi, Norito Kawakami, Akihito Shimazu, Akizumi Tsutsumi

**Affiliations:** 1 Department of Public Health, Kitasato University School of Medicine, Sagamihara, Japan; 2 Department of Mental Health, Graduate School of Medicine, The University of Tokyo, Tokyo, Japan; 3 Faculty of Policy Management, Keio University, Fujisawa, Japan

**Keywords:** Occupational stress, Turnover, Workers, Administrative data

## Abstract

**Background:**

Although several studies have examined the association between occupational stress and turnover, these studies relied on cross-sectional designs, subjects’ self-report, healthcare workforce, or small sample sizes. This study aimed to confirm whether occupational stress increases the risk of turnover in a large-scale prospective cohort study using actual turnover data from company records.

**Methods:**

The participants were 3892 male and 5765 female employees aged 20–49 years in a financial service company. We followed them from October 2012 until April 1, 2016 and used company records to identify employees who resigned. We identified employees with high and low stress using the Brief Job Stress Questionnaire. Hazard ratios for turnover in high-stress employees were estimated using Cox proportional hazards models, and population attributable risks were calculated separately for men and women.

**Results:**

During 11,475,862 person-days, 122 men and 760 women resigned. After adjustment for age, length of service, job type, and position, the hazard ratios (95% confidence intervals) for turnover in high-stress employees were 2.86 (1.74–4.68) for men and 1.52 (1.29–1.78) for women. The corresponding population attributable risks for high stress were 8.2% for men and 8.3% for women. The component scores, i.e., job stressors, psychological/physical stress response, workplace social support, and job strain (the combination of high job demands and low job control) were also significantly associated with turnover (*p* < 0.05).

**Conclusions:**

Occupational stress increases the risk of actual turnover. Measures to prevent occupational stress may be useful to prevent employee turnover.

## Background

Employee turnover is a serious issue faced by many organizations worldwide. Not only is turnover costly in terms of recruiting and training new employees [1], but it can also be costly in terms of reduction in profits through reduced team performance and service levels [2–4]. Preventing turnover is a critical management issue. In some cases, turnover is also costly for workers, as they give up career or interpersonal connections at their previous place of employment [5].

Several studies have reported that occupational stress increases the risk of turnover. However, most studies did not investigate actual turnover, but instead addressed turnover intentions [6]. Further, even studies that tracked actual turnover [7–17] relied on subjects’ recalled self-report [8–11, 13, 14, 17], not by administrative data, which limit the reliability of the data obtained. In addition, more than half of these studies that tracked actual turnover targeted healthcare workers [11–16], whose turnover rates are notoriously high due to its workforce shortage in many developed countries [6]. Further studies in the sector other than health care are needed. Finally, their sample size was comparatively small (less than 1000) [8, 9, 11–14, 16].

In Japan, the turnover rate has been comparatively low due to unique labor market characteristics [18, 19]. Japan has relied on life-time employment for a long time until 1990s, and then rapidly changed into a contract-based labor market after an economic recession, while labor market mobility remained less flexible. Thus, Japanese workers, particularly male permanent workers with a breadwinner role, tend not to leave the company unless for some extraordinary reason [20]. We aimed to investigate the association between occupational stress and actual turnover via a large-scale prospective cohort study. Towards this goal, we used actual turnover data from the human resources records of employees in a company.

## Methods

### Study design and setting

We conducted a prospective cohort study using human resources records of employees in a financial services company listed on the major stock exchange market. This company conducts a stress check examination annually to maintain and improve employees’ health and safety since 2010.

### Participants

**Fig. 1:**
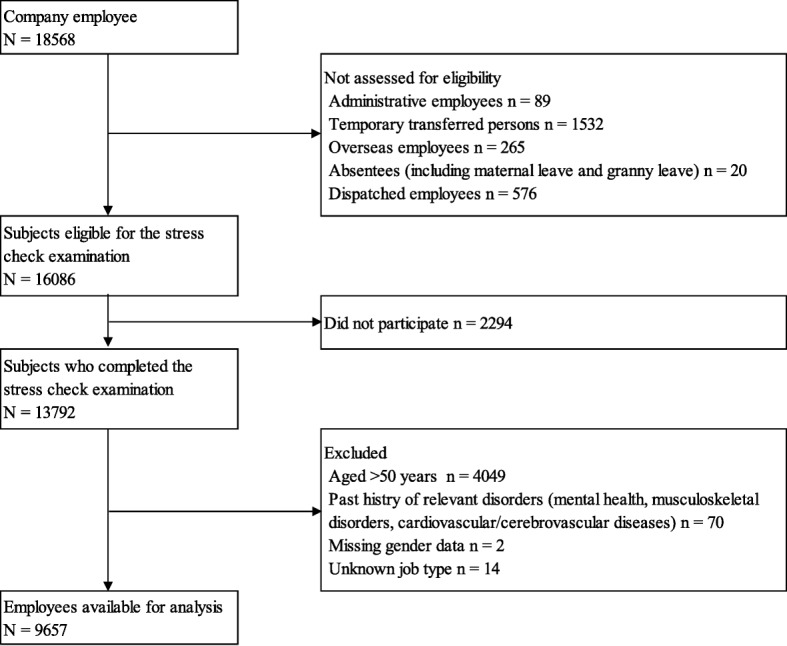
Recruitment and follow-up flow diagram

The participants were male and female employees aged 20–49 years in a financial service company. Of the 16,086 employees eligible for the stress check examination (Fig. 1), 13,792 employees completed the BJSQ between October 2012 and November 2012, yielding a response rate of 85.7%. At baseline, we excluded 4049 employees aged 50 and older because they might leave due to (early) retirement during follow-up. We also excluded 70 employees who had a disease history according to past sick pay records (mental disorder, musculoskeletal disorder, cardiovascular diseases, and cerebrovascular diseases) because they might leave due to relapse. Further, we excluded 2 employees with missing gender data, and 14 employees with unknown job type. Thus, a total of 9657 employees (3892 men, 5765 women; aged 20–49 years) were followed until April 1, 2016. The company provided anonymous data. Informed consent was obtained from participants using the opt-out method.

### Variables

#### Outcome

We identified the employees’ turnover date using the human resources records of employees in a company. Generally, employee turnover refers to the voluntary or involuntary departure of employees from their organizations [21]. Voluntary turnover is initiated by the employee’s own ambitions or dissatisfaction with work and employment conditions, while involuntary turnover is initiated by the organization (e.g., layoffs and dismissals) [21]. All turnover cases in this study were voluntary.

#### Stress profile

We used the construct of occupational stress adopted by The Stress Check Program, which is a new occupational health policy launched by the Japanese government on December 1, 2015. This policy targets the primary prevention of mental health problems by annually monitoring and screening workers with high stress at the workplace and is mandatory for workplaces with 50 or more employees [22].

The occupational stress adopted by the Stress Check Program manual include the following three components: (1) job stressors, (2) stress responses, and (3) social supports [23]. Although each workplace can choose any questionnaire to assess these three components, the Stress Check Program manual recommends using the Brief Job Stress Questionnaire (BJSQ). The BJSQ is a 57-item questionnaire that assesses job stressors, stress responses, and social supports as buffering factors based on the NIOSH Job Stress Model [24, 25]. This questionnaire assesses nine aspects of job stressors: quantitative job overload (three items), qualitative job overload (three items), physical demands (one item), job control (three items), skill utilization (one item), interpersonal conflict (three items), poor physical environment (one item), suitable jobs (one item), and meaningfulness of work (one item). The items for job control, skill utilization, suitable jobs, and meaningfulness of work were reversed items. Stress response included the following six aspects: vigor (three items), irritation (three items), fatigue (three items), anxiety (three items), depression (six items), and physical complaints (11 items). Social supports included the following three supports: supervisor support (three items), coworker support (three items), and support from family and friends (three items). This questionnaire also assesses job satisfaction and life satisfaction (one item for each). The answers were provided on a four-point Likert scale (1 = *Not at all*, 2 = *Somewhat*, 3 = *Moderately so*, and 4 = *Very much so* for job stressors; 1 = *Almost never*, 2 = *Sometimes*, 3 = *Often*, and 4 = *Almost always* for stress responses; and 1 = *Not at all*, 2 = *Somewhat*, 3 = *Very much*, and 4 = *Extremely* for social supports). Cronbach’s α coefficients were 0.74, 0.69, 0.94, and 0.87 for the job demand, job control, stress response, and social support scale, respectively. All BJSQ scales have been proven to have acceptable or high levels of internal consistency reliability and factor-based validity [24].

The Stress Check Program manual proposes criteria for defining *high-stress* employees based on the BJSQ [22]. The NIOSH Job Stress model postulates job stressors are treated as predictors, stress responses as mediators, and social supports as effect modifiers. However, in our study, according to the instruction of the Stress Check Program, we constructed ‘high stress’ based on the combination of the three components (job stressors, stress responses, and social support). High stress is defined as the higher level of stress response (criterion A) or having a moderate level of stress response, together with having higher job stressors or lower workplace social support (criterion B). To calculate the score of job stressor, we simply summed the subscale scores of quantitative job overload, qualitative job overload, physical demands, job control, skill utilization, interpersonal conflict, poor physical environment, suitable jobs, and meaningfulness of work. In a similar way, the scores of stress response and social support were calculated. The scores for stress response and the sum of job stressor and social support ranged from 29 to 116 and from 26 to 104, respectively. The cutoff points proposed by the Stress Check Program manual were 77 for the stress response score (criterion A), 76 for the job stressor and social support score, and 63 for the stress response score (criterion B). The criteria have been proven to show good predictive validity for sickness absence [26].

#### Covariates

Covariates included gender, age (20–29, 30–39, and 40–49 years), length of service (0–4, 5–9, ≥10 years), job type (sales, complaint service, or administrative), and position (staff, manager, or temporary employee).

### Statistical analysis

We analyzed data separately for men and women because of significant difference in job type and position between genders. First, the baseline characteristics were described as numbers (percentage) and were compared between high-stress employees and others using chi-square tests. Second, a Kaplan-Meier curve was generated to compare the cumulative incidences of turnover between high-stress employees and others. Third, Cox’s proportional hazard regression analysis was used to investigate the association between stress profiles and onset of turnover. Hazard ratios (HRs) were estimated first after adjusting for age, and then additionally adjusting for length of service, job type, and position. Finally, we estimated the population-attributable risk (PAR) for high stress. The PAR is the fraction of all cases of turnover in a population due to exposure to occupational stress. The PAR percent was calculated as: (HR-1)*p/(1 + [HR-1]*p), where p is the prevalence of high stress in the total population at baseline and HR is the hazard ratio for incident turnover for high stress versus others. We adjusted the PAR estimates for covariates in a similar way to the corresponding Cox models for HRs.

We also conducted three sensitivity analyses. First, we separately tested the stress profiles defined by criteria A and B. Second, we tested the association between occupational stress and onset of turnover using each score for psychological and physical stress response (range 29 to 116), job stressors (17 to 68), and workplace social support (9 to 36) as predictors instead of the stress profiles defined by the Stress Check Program manual. Finally, we evaluated occupational stress by the job demands-control (JDC) model [27] for comparison with previous studies using the JDC model [8, 9, 12]. This model explains occupational stress as an interaction between job demands and job control such that the strongest physiological responses are expected in situations involving high job demands and low job control [27]. Job demands and job control were measured by quantitative job overload and job control subscales of the BJSQ, respectively. Each subscale score ranged 3 to 12. Job strain was defined according to a procedure often used in other studies; individuals in the upper quartile of a job strain ratio (demand score divided by control score) are defined as being exposed [28]. We then tested the association between job strain and onset of turnover. All statistical tests were two-sided, with a 5% significance level. All analyses were conducted using SAS version 9.3 for Windows (SAS Inc., Cary, NC, USA).

## Results

Table 1 shows the baseline characteristics by stress profiles. High-stress was prevalent in 4.8% of male employees. Male high-stress employees were older and less likely to be a manager compared to other employees. Meanwhile, high-stress employees was prevalent in 17.5% of female employees. Female high-stress employees were older, had worked in the company for a longer period, and more likely to work in complaint service sectors.

**Table 1:** Baseline characteristics by gender and stress profiles as defined by the Brief Job Stress Questionnaire

	Men (*n* = 3892)					Women (*n* = 5765)				
	High stress		Others		*p*-value	High stress		Others		*p*-value
No. of participants	187		3705			1007		4758		
Age, years										
20–29	25	(13.4)	748	(20.2)	0.004	477	(47.4)	2482	(52.2)	0.005
30–39	72	(38.5)	1042	(28.1)		291	(28.9)	1339	(28.1)	
40–49	90	(48.1)	1915	(51.7)		239	(23.7)	937	(19.7)	
Length of service, years										
0–4	31	(16.6)	596	(16.1)	0.221	375	(37.2)	1969	(41.4)	0.018
5–9	36	(19.3)	548	(14.8)		316	(31.4)	1306	(27.5)	
≥ 10	120	(64.2)	2561	(69.1)		316	(31.4)	1483	(31.2)	
Job type										
Sales	93	(49.7)	2130	(57.5)	0.097	510	(50.7)	2644	(55.6)	< 0.001
Claims service	42	(22.5)	662	(17.9)		378	(37.5)	1251	(26.3)	
Administrative	52	(27.8)	913	(24.6)		119	(11.8)	863	(18.1)	
Position										
Staff	132	(70.6)	2231	(60.2)	< 0.001	922	(91.6)	4436	(93.2)	0.007
Manager	39	(20.9)	1413	(38.1)		4	(0.4)	45	(1.0)	
Temporary employee	16	(8.6)	61	(1.7)		81	(8.0)	277	(5.8)	

Values are presented as n (%). Variables were compared using chi-square tests

**Fig. 2:**
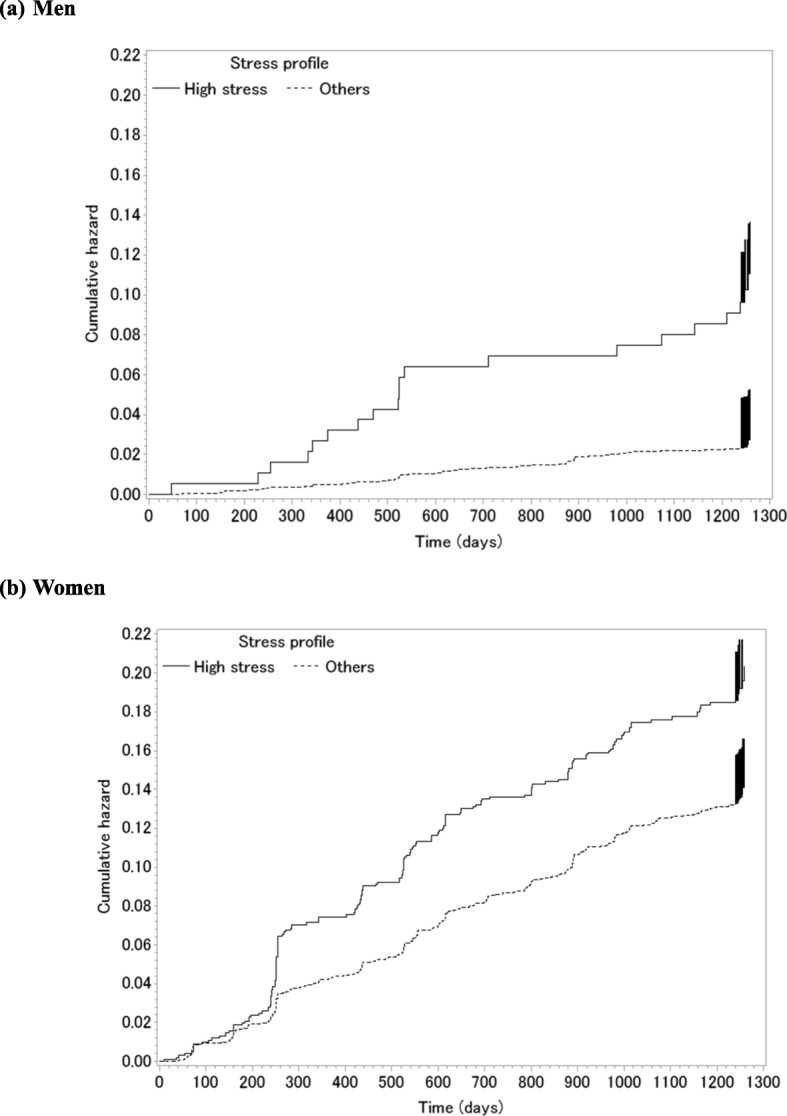
Cumulative hazard risks for turnover in (a) high-stress men and (b) high-stress women

During 11,475,862 person-days, 122 men and 760 women left the company. The Kaplan-Meier plots showed high-stress employees were more likely to leave the company than others for both men and women (Fig. 2). The progression of turnover was more constant among women than men.

Table 2 shows the results of Cox’s proportional hazard regression analysis and PAR for high stress. The age-adjusted HR for incident turnover in high-stress men showed a 4-fold higher risk. After additional adjustment for length of service, job type, and position, the risk for men decreased to 2.86 (95% confidence interval [CI]: 1.74–4.68). The age-adjusted HR in high-stress women was 1.54. The HR for women did not change after adjustment of all covariates (HR: 1.52, 95% CI: 1.29–1.78). PAR for high stress that was calculated with the observed HR of fully-adjusted model was 8.2% for men and 8.3% for women.

**Table 2:** Associations between stress profiles as defined by the Brief Job Stress Questionnaire (BJSQ) and the incidence of turnover

	Person-days	Cases	Rate/1000person-days	HR (95% CI)						
				Crude model		Age-adjusted model^a^		Fully-adjusted model^b^		%PAR^c^
Men (*n* = 3892)										
High stress	222,908	21	0.09	4.25	(2.65, 6.80)^*^	4.37	(2.72, 7.00)^*^	2.86	(1.74, 4.68)^*^	8.2%
Others	4,591,544	101	0.02	1.00		1.00		1.00		
Women (*n* = 5765)										
High stress	1,124,502	195	0.17	1.45	(1.24, 1.70)^*^	1.54	(1.32, 1.81)^*^	1.52	(1.29, 1.78)^*^	8.3%
Others	5,536,908	665	0.12	1.00		1.00		1.00		

*HR* Hazard ratio; *CI* confidence interval; *PAR* Population-attributable risk. *p < 0.05

^a^Adjusted for age

^b^Adjusted for age, length of service, job type, and position

^c^PAR was calculated with the observed HR of fully-adjusted model

The prevalence of high-stress as defined by criterion A was 3.8% for men and 15.9% for women. Meanwhile, its prevalence as defined by criterion B was 2.0% for men and 6.1% for women. We found almost the same level of HRs and PARs for criterion A as for high stress defined by a combination of criteria A and B. However, the level of HRs and PARs for criterion B was lower than that for high stress defined by a combination of criteria A and B (Table 3).

**Table 3:** Associations between stress profiles based on criterion A or B and the incidence of turnover

	HR (95% CI)						
	Crude model		Age-adjusted model^a^		Fully-adjusted model^b^		%PAR^c^
Criterion A^e^							
Men (*n* = 3892)							
High stress	4.80	(2.94, 7.83)^*^	4.94	(3.02, 8.07)^*^	3.32	(1.99, 5.56)^*^	8.1%
Others	1.00		1.00		1.00		
Women (*n* = 5765)							
High stress	1.50	(1.27, 1.76)^*^	1.57	(1.33, 1.84)^*^	1.54	(1.31, 1.82)^*^	7.9%
Others	1.00		1.00		1.00		
Criterion B^e^							
Men (*n* = 3892)							
High stress	3.13	(1.46, 6.72)^*^	3.26	(1.52, 7.00)^*^	1.95	(0.89, 4.26)^*^	1.9%
Others	1.00		1.00		1.00		
Women (*n* = 5765)							
High stress	1.22	(0.93, 1.58)	1.45	(1.11, 1.88)^*^	1.39	(1.07, 1.80)^*^	2.3%
Others	1.00		1.00		1.00		

*HR* Hazard ratio; *CI* confidence interval; *PAR* Population-attributable risk. *p < 0.05

^a^Adjusted for age

^b^Adjusted for age, length of service, job type, and position

^c^PAR was calculated with the observed HR of fully-adjusted model

^e^Criterion A is defined as the highest level of stress response as measured via the Brief Job Stress Questionnaire (cutoff 77) and criterion B is defined as a moderate or higher level of stress response (cutoff 63), along with having the highest job stressors (or lowest social support in the workplace) (cutoff 76), according to the Stress Check Program manual. It should be noted that there is overlap in the distribution of criteria A and B

In the fully-adjusted analyses where each score of psychological and physical stress response, job stressor, and workplace social support was entered separately, the HR of incident turnover was statistically significant for each of these predictors both among men and women. Among men, the HR per 1-point increase of the score was 1.06 (95% CI: 1.03–1.09) for psychological and physical stress response, 1.03 (95% CI: 1.02–1.04) for job stressors, and 1.08 (95% CI: 1.04–1.12) for lack of workplace social support. Among women, the HR per score unit was 1.03 (95% CI: 1.02–1.05) for psychological and physical stress response, 1.01 (95% CI: 1.01–1.02) for job stressors, and 1.01 (95% CI: 1.00–1.02) for lack of workplace social support.

When job strain was entered as a predictor in the fully-adjusted model, the HR of incident turnover was statistically significant for job strain both among men (HR: 1.55, 95% CI: 1.06–2.27) and women (HR: 1.29, 95% CI: 1.12–1.50).

## Discussion

This cohort study showed significant prospective associations between occupational stress and actual turnover in a Japanese working population, where the job mobility is markedly less frequent than that in Western countries. Male and female high-stress employees had approximately 3 times higher and 1.5 times higher risk of turnover, respectively, compared with their low-stress counterparts. Although the magnitude of the effect of high stress on turnover was smaller in women than in men, the impact of high stress in this population (i.e., the size of the PAR) was almost similar (8%) between genders because the high stress was more prevalent in women (17.5%) than in men (4.8%).

Similar results from previous studies conducted in China and European countries, including Denmark, the Netherlands, and Sweden, were obtained despite differences in social and cultural contexts. These studies indicated that adverse psychosocial work environment evaluated according to workplace bullying, the JDC model [27], and the effort-reward imbalance model [29] and stress responses such as depression and anxiety were associated with the incidence of turnover [7–17, 30]. We expanded the generalizability of these findings by using administrative outcome data and recruiting a general worker population whose turnover rate was comparatively low.

Our results from supplemental analyses based on the JDC model confirmed that job strain was associated with the incidence of turnover, consistent with a previous study [8]. However, the magnitude of HR for high stress was higher than that for job strain both among men and women. This suggests that the combination of job stressors and stress response may be more predictive of turnover than limited job stressors only.

Our study also illustrated gender differences in the magnitude of the effect of high stress on turnover, and the HR was smaller for women than men. Such gender differences have been found in previous studies [8]. This may be due to the social norms of gender roles, that is, men as breadwinner and women as caregiver. Some women may leave the company due to family roles such as housekeeping and childrearing [31]. Thus, the association between occupational stress and turnover may have been weaker among women than among men.

Men were less likely to leave the company than women, which might reflect the still prevailing Japanese male-breadwinner model [20]. Turnover rates spiked during the first 2 years then plateaued among men, whereas the progression was constant among women. The different patterns of the turnover may be due to the difference in job types between genders. Most men worked as sales personnel selling insurance, while more women were engaged in the complaint service department of the insurance company. Compared to the complaint service department where the work is more predictable and fixed, sales is more flexible and the job responsibilities are likely to change according to the customers and the insurance products, and the stress level may have changed during the follow-up.

Our study suggested that reducing occupational stress could help prevent turnover. Previous studies suggested that improving the work environment and providing stress management skills to high-stress workers effectively reduce occupational stress [32–34]. Thus, organizations should consider improving the work environment and providing stress management training to prevent turnover [32]. However, unmeasured other factors, probably including management style, career advancement, and pay/benefits [6], should also be considered to prevent turnover.

This study has some limitations. First, our study was based on a convenient sample, which mainly comprised white-collar workers engaged in sales, complaint service, and administration of a financial company. Thus, the generalizability of the results is limited. Second, we lacked data on potential confounders such as working hours and work-life balance. Third, although all turnover cases in this study was voluntary turnover, we lacked data on the reasons for turnover. Despite these limitations, our study had several important strengths, including its large sample size, the use of actual turnover data from company records, and longitudinal design. In addition, our study shows that the criteria for defining high stress set by the Stress Check Program manual have an advantage in that it is simple and practical for identifying individuals at risk of turnover.

## Conclusions

The results of this cohort study indicate that occupational stress is associated with a higher risk of turnover among both male and female Japanese employees. Future studies should replicate this association in other population and examine the effectiveness of reducing occupational stress on turnover reduction among employees.

## Data Availability

The datasets analyzed during the current study are not publicly available due to privacy concerns and institutional policy.

## Abbreviations

*BJSQ*: Brief Job Stress Questionnaire
*HRs*: Hazard ratios
*JDC*: Job demands-control
*PAR*: Population-attributable risk
